# Design of a Lorentz Force Magnetic Bearing Group Steering Law Based on an Adaptive Weighted Pseudo-Inverse Law

**DOI:** 10.3390/s25103242

**Published:** 2025-05-21

**Authors:** Chenyu Wang, Lei Li, Weijie Wang, Yanbin Zhao, Baiqi Li, Yuan Ren

**Affiliations:** 1 The Graduate School, Space Engineering University, Beijing 101416, China; 506416939@hgd.edu.cn (C.W.); baiqi_li@hgd.edu.cn (B.L.); 2 The Department of Astronautics Science and Technology, Space Engineering University, Beijing 101416, China; wangweijie@126.com (W.W.); renyuan_821@aliyun.com (Y.R.); 3 Shanghai Institute of Satellite Engineering, Shanghai 200240, China; 15900908925@139.com

**Keywords:** Lorentz force magnetic bearings, adaptive weighted pseudo-inverse, steering law design, genetic optimization algorithm

## Abstract

Aiming at the high-precision torque output and saturation singularity avoidance problems in Lorentz force magnetic bearing (LFMB) swarms for magnetic levitation spacecraft, this study designs a manipulation law based on an adaptive weighted pseudo-inverse law. The system monitors each magnetic bearing’s working state in real time using high-precision position and current sensors. As the key input for the adaptive weighted pseudo-inverse control law, the sensor data’s measurement accuracy directly determines torque distribution effectiveness and attitude control precision. First, considering electromagnetic back-EMF effects, individual LFMB dynamics are modeled via the equivalent magnetic circuit method, with working principles elucidated. Subsequently, saturation coefficients for LFMB swarms are designed. Incorporating spacecraft maneuvering requirements, a genetic optimization algorithm establishes the optimal mounting configuration under task constraints. Considering the LFMB swarm configuration characteristics, this study proposes an adaptive weighted pseudo-inverse maneuvering law tailored to operational constraints. By designing an adaptive weighting matrix, the maneuvering law adjusts each LFMB’s torque output in real time, reducing residual saturation effects on attitude control speed and accuracy. Simulation results demonstrate that the proposed mounting configuration and adaptive weighted pseudo-inverse maneuvering law effectively mitigate saturation singularity’s impact on attitude control accuracy while reducing total energy consumption by 22%, validating the method’s effectiveness and superiority.

## 1. Introduction

Currently, complex space missions, such as interplanetary laser communication and deep space exploration, have increasingly high requirements for the pointing accuracy and stability of satellite attitude loads. In traditional satellites, the flywheel and control torque gyroscope momentum exchange control the satellite’s attitude. However, for the flywheel, control torque gyroscope, and other attitude control actuators using a mechanical bearing support mode, there are mechanical friction and vibration problems, and it has been difficult to meet these needs. In this context, magnetic levitation platform technology shows great potential for application by virtue of its non-contact and long-life characteristics. This new spacecraft attitude control technology based on a magnetic levitation mechanism has become a hot research topic at home and abroad.

The new spacecraft attitude control technology of magnetic levitation mechanisms is divided into a system level and component level, where the component level is used to replace the traditional mechanical actuator with the new magnetic levitation flywheel and control moment gyro. A magnetically suspended flywheel energy storage system (FESS) realizes frictionless rotor suspension through magnetic bearing technology, which significantly reduces standby loss and improves power density, providing efficient and fast energy conversion and stable support for uninterruptible power supply (UPS) [1]. Germany’s Teldix company developed a 30 Nms Lorentz force configuration for a magnetic levitation flywheel, and this rotor can realize five degrees of freedom levitation [2]. China’s National University of Defense Technology (NUDT) developed a hybrid configuration of a maglev flywheel on the basis of the first two configurations, combining the advantages of their respective configurations, and this flywheel can achieve the deflection angle and output 3 N·m deflection torque [3]. At the Beijing University of Aeronautics and Astronautics, Academician Fang’s team used research based on the development of a magnetic levitation control moment gyroscope to achieve high-precision attitude control of large remote sensing satellites [4,5]. In 2024, at the Aerospace Engineering University, Professor Ren’s team developed the Lorentz force magnetic levitation universal stabilization platform to solve the start of the vibration of the traditional mechanical two-axis rotary table interference, while the existing magnetic levitation satellites exist in the payload attitude motion and the platform attitude movement of mutual constraints bottleneck problem [6]. In summary, the new magnetic levitation actuator, compared to the traditional actuator, reduces the resulting vibration and improves the attitude-pointing accuracy and stability of the spacecraft, but only by reducing the vibration can the spacecraft and actuator momentum exchange process be carried out to isolate the vibration completely.

The spacecraft system ensures vibration isolation between the spacecraft and the payload by means of magnetic levitation techniques. Vibration isolation techniques are categorized into active and passive vibration isolation, and spacecraft usually adopt active vibration isolation techniques due to the periodic vibration sources of their moving parts [7]. Spaceborne high-precision optical systems put forward strict requirements for the on-orbit micro-vibration environment, and the lack of decoupling performance of traditional Stewart platforms directly affects the imaging quality. The latest research shows that by establishing a comprehensive parametric dynamic model and optimizing key structural parameters, the vibration isolation performance of the platform can be significantly improved, providing a more stable working environment for optical loads [8].

**Figure 1:**
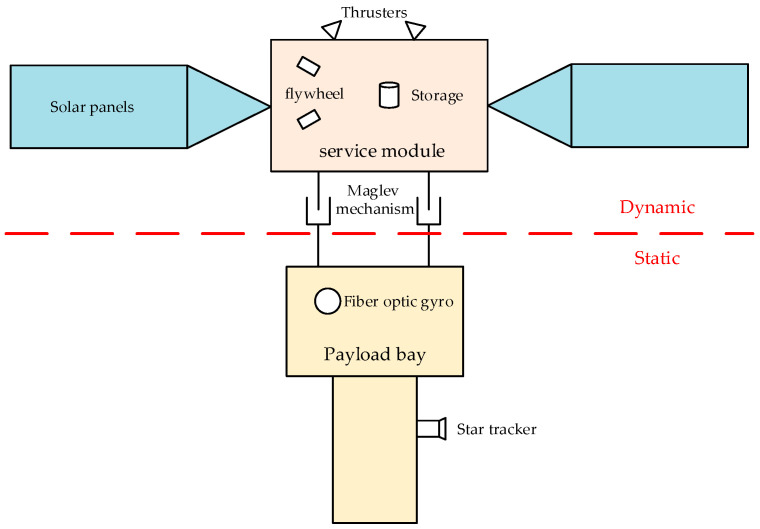
Schematic diagram of the structure of the DFP satellite.

Subsequently, due to the superiority of its vibration isolation effect, many vibration isolation devices based on the Stewart platform have been designed, but actuators using mechanical bearings cannot achieve complete isolation of vibration. The concept of a disturbance-free payload (DFP) satellite design was proposed by Lockheed in 2003 [9,10,11]. A DFP satellite is designed as being relatively independent and organically combines two compartments through a non-contact magnetic levitation mechanism, while the two components are connected by the non-contact signal channel, non-contact relative position sensor, non-contact electromagnetic magnetic levitation mechanism, and the flexible magnetic levitation mechanism. The two compartments are organically connected through the contactless signal channel, contactless relative position sensor, contactless electromagnetic magnetic levitation mechanism, and flexible cable to realize energy and information transmission so that the vibration and interference of the platform module will not be transmitted to the payload module, and the overall structure is shown in Figure 1 [12]. In recent years, in order to meet the stringent requirements of modern satellites for ultra-high stability and ultra-high precision, some aerospace colleges and research institutes have also initiated research on DFP satellites [13,14]. In 2014, the Shanghai Satellite Engineering Research Institute put forward the “double super” satellite platform to break the traditional solid connection design, using a non-contact, high-precision, non-time-delayed magnetic levitation actuator to realize the separation of the load (module) with only quiet parts and the platform (module) with movable parts, thus completely eliminating the influence of micro-vibrations [15,16]. In China, Kong Yongfang et al. proposed some reliable control application methods on interference-free payloads to further improve the attitude control accuracy of DFP spacecraft [17,18]. In the magnetic levitation system, high-precision sensor networks are the basis for non-contact control. The position sensor measures the relative displacement between the platform cabin and the load cabin in real time, and the current sensor monitors the working state of each magnetic bearing. These sensing data constitute the feedback closed loop of the control system [19].

Magnetic levitation actuators are magnetic bearings based on the Lorentz force principle, and the combined operation of multiple magnetic bearings provides three-axis translational control force and three rotational control moments, in which the control allocation method plays a key role in the combined operation of the magnetic bearing group, which not only optimizes the allocation of the control force but also ensures that the actuator constraints are satisfied, improves the reliability and robustness of the system, and simplifies the control design process [20].

Currently, more widely used actuator control allocation methods include the generalized inverse method, including the pseudo-inverse method and its derivatives (the weighted pseudo-inverse method, redistributive pseudo-inverse method, etc.), as well as the chain method, etc. Zhang Wei et al. used the pseudo-inverse method to establish a control matrix to solve the output force of a single magnetic bearing through the installation layout and could not control the oversaturation of the magnetic bearing [16,20]. Zhang Jian et al. considered different specifications of magnetic bearings using different control efficiency matrices to solve the problem; although different control efficiency matrices are considered, it is also impossible to avoid oversaturation [21]. Aiming at the problem of control allocation, Wang Zhihui et al. used the weighted pseudo-inverse method to control and allocate multiple actuators in the distributed propulsion configuration aircraft, which improved the control accuracy [22]. A weighted pseudo-inverse control allocation method was used in the literature [22] for redundant actuators of a tilt-rotor aircraft to achieve full utilization of the redundant mechanisms.

Aiming at the above problems, this paper proposes a magnetic bearing group manipulation law based on weighted pseudo-inverse methods. An adaptive function is designed through the saturation situation of the Lorentz force magnetic bearing output, and the weighting matrix weights are adjusted and assigned according to the adaptive function, which can make each magnetic bearing output relatively uniform to avoid collision. Furthermore, an adaptive sliding mode controller is designed to compensate for the torque error, which improves the pointing accuracy and stability of the attitude control of the magnetic levitation spacecraft.

## 2. Lorentz Force Magnetic Bearing Principle Analysis and Dynamic Modeling

### 2.1. Analysis of the Working Principle of Magnetic Bearings

**Figure 2:**
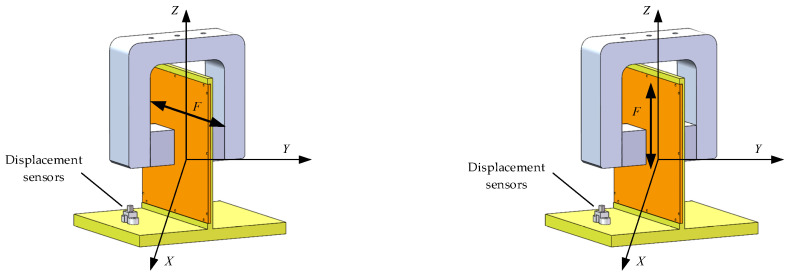
Structural diagram of Lorentz force magnetic bearings.

The working principle of the Lorentz force magnetic bearing is based on the force of a magnetic field on the charged particles in motion; as its output is linear with the current, there is no current negative stiffness, and the control accuracy is high. It is mainly composed of a stator, rotor, and coil. The stator is a fixed magnet, the rotor is a movable iron core, and the coil takes the form of a printed circuit board engraved on both sides of the core. When the coil is energized, the magnetic field produced by the current interacts with the magnetic field of the stator to produce a torque that moves the rotor along its axis. This force can be expressed by the equation $F=NBIL_{x}$ where the force F is acting on the rotor, N is the number of coil ties, B is the current in the coil, I is the flux density, $L_{x}$ is the length of the coil, and the magnetic bearing structure is shown in Figure 2.

### 2.2. Dynamics Modeling

**Figure 3:**
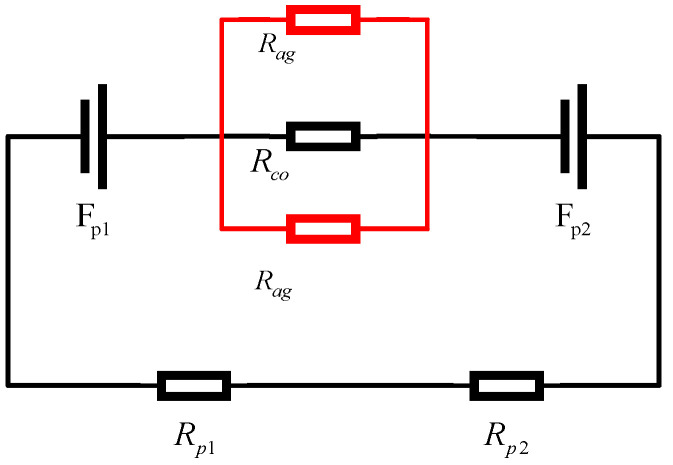
Magnetic bearing equivalent magnetic circuit diagram.

Assuming that the magnetic bearing only considers the reluctance of the operating air gap and does not consider the reluctance of the iron core, in the case of neglecting the eddy current loss, according to the structure diagram of the magnetic bearing, the equivalent magnetic circuit diagram of magnetic bearing can be obtained as shown in Figure 3, in which $F_{p1}$, $F_{p2}$ represent the magnetomotive force generated by the magnetic poles of the magnetic bearing, the magnetomotive force corresponds to the permanent magnet reluctance $R_{p1}$,$R_{p2}$, the reluctance of the air gap that passes through the inner and outer magnet steel air gaps is $R_{co}$, and the magnetic leakage reluctance of the air gap is $R_{ag}$. The permanent magnetic circuit can generate a constant uniform vertical magnetic field B in the air gap along the axial direction, in which the magneto-kinetic potential of the magnet steel ring is linearly related to the toughened length of the magnet steel and all the magnetoresistances are serially connected. The equivalent magnetic circuit established is shown in Figure 3.

The mathematical expressions for the main flux m and magnetic induction B of the coil can be obtained from Ohm’s law for magnetic circuits, as follows:

(1) $$\phi_{m}=\frac{\sum_{i=1}^{2}F_{pi}}{\sum_{i=1}^{2}R_{pi}+R_{co}}$$

(2) $$B=\frac{\phi }{S}$$

Substituting (1) into (2), Equation (3) is as follows:

(3) $$B=\frac{H_{c}(l_{p1}+l_{p2})}{(\frac{l_{1}}{S_{1}\mu_{0}\mu_{r}}+\frac{l_{2}}{S_{2}\mu_{0}\mu_{r}}\frac{\delta }{\mu_{0}S}+\frac{l_{p1}}{S_{p1}\mu_{0}\mu_{r}}+\frac{l_{p2}}{S_{p2}\mu_{0}\mu_{r}})S}$$

Table 1 shows the definitions of symbols in Formulas (3)–(6).


(4) $$F=NBIL_{x}$$

(5) $$\left[\begin{array}{l} F_{y}\\ F_{z} \end{array}\right]=\left[\begin{array}{l} I_{z}\\ I_{y} \end{array}\right]N\left(\frac{L_{M}}{A_{o}}+\frac{L_{m}}{A_{i}}\right)\times \frac{H_{c}(l_{p1}+l_{p2})}{\frac{l_{1}}{S_{1}\mu_{0}\mu_{r}}+\frac{l_{2}}{S_{2}\mu_{0}\mu_{r}}\frac{\delta }{\mu_{0}S}+\frac{l_{p1}}{S_{p1}\mu_{0}\mu_{r}}+\frac{l_{p2}}{S_{p2}\mu_{0}\mu_{r}})S}$$

Under normal operating conditions, the magnetization length, equivalent reluctance, and equivalent cross-sectional area corresponding to the internal and external flux remain basically unchanged, and it can be assumed that the magnetic bearing force and coil current are linearly related, so the above equation is equivalent to the following Equation (6):

(6) $$\left[\begin{array}{c} F_{y}\\ F_{z} \end{array}\right]=K\left[\begin{array}{c} I_{z}\\ I_{y} \end{array}\right]$$

where K is the coil current stiffness factor.

In electromagnetism, when an energized coil moves through a magnetic field and cuts a line of magnetic inductance, an electromotive force is generated in that coil according to Faraday’s law of electromagnetic induction, which is often referred to as the induced electromotive force or counter-electromotive force.

**Figure 4:**
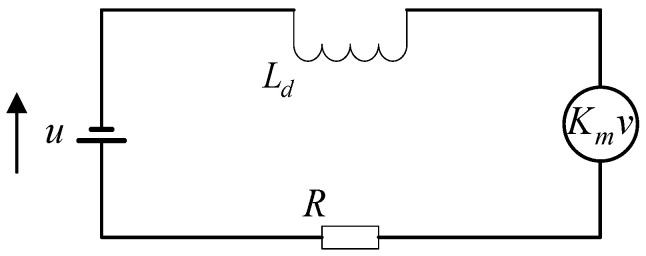
Magnetic bearing circuit diagram.

According to the circuit schematic of a magnetic bearing in Figure 4, Equation (7) is as follows:

(7) $$E=-L_{d}\frac{dI}{dt}-K_{m}\frac{dL_{w}}{dt}$$

where the first item is the self-inductance electromotive force (blocking current change), and the second item is the motion electromotive force (related to the cutting magnetic induction line speed). Equation (8) is as follows:

(8) u−E=I⋅R

Table 2 shows the definitions of symbols in Formulas (7) and (8).

When the disturbing force caused by back electromotive force is taken into account, the dynamic equation of the magnetic bearing is as follows:

(9) $$m\frac{d^{2}L_{m}}{dt^{2}}=F_{c}+F_{m}+F_{f}$$

where m is the mass of the magnetic bearing rotor; $F_{c}$ is the force output from the controller; $F_{m}$ is the disturbance force caused by the reverse electromotive force; and $F_{f}$ is another resistance, such as air.

Since this magnetic bearing is used in aerospace applications, i.e., it can be neglected, the kinetic equation is as follows:

(10) $$m\frac{d^{2}L_{m}}{dt^{2}}=F_{c}+F_{m}$$

The force output $F_{c}$ by the controller and the disturbance force $F_{m}$ caused by the back electromotive force comprise the driving force F output by the magnetic bearing.

(11) F=KI

By applying the Laplace transform of Equations (8) and (11), it yields the following Equations (12) and (13):

(12) $$U(s)+L_{d}I(s)+K_{m}L_{w}(s)=I(s)\cdot R$$

(13) F(s)=KI(s)

Substituting Equation (12) into (13) yields the following Equation (14):

(14) $$F(s)=\frac{KU(s)+KK_{m}L_{w}(s)}{R-L_{d}}=F_{c}+F_{m}$$

Considering that the inductance $L_{d}$ is generally negligible, Equation (15) is as follows:

(15) $$F_{m}=\frac{KK_{m}L_{w}\left(s\right)}{R}=K_{b}L_{w}\left(s\right)$$

where Equation (16) is as follows:

(16) $$K_{b}=\frac{KK_{m}}{R}$$

Then, substituting Equation (15) into Equation (14) and performing the inverse Laplace transform yields the following Equation (17):

(17) $$F=KI+K_{b}\frac{dL_{w}}{dt}$$

The final kinetic equation for the magnetic bearing single degree of freedom is derived as follows:

(18) $$m\frac{d^{2}L_{w}}{dt^{2}}=KI+K_{b}\frac{dL_{w}}{dt}$$

From the characteristic root analysis, it can be observed that the magnetic bearing system possesses a positive characteristic root, demonstrating inherent instability characteristics in the absence of a control input. To address this property, this study implements a sliding mode controller in the case simulations of Section 4, leveraging its strong robustness to suppress system divergence tendencies and thereby guarantee closed-loop stability. During system modeling, particular attention is given to investigating the impacts of position sensor measurement noise and current sensor zero drift on control precision. The sensing unit design employs a differential inductance structure for position detection, achieving a resolution of 0.1 μm and a bandwidth of 500 Hz to precisely capture rotor micro-displacements. Current monitoring based on the closed-loop Hall principle provides high-fidelity current feedback for back-EMF compensation, exhibiting a linearity index superior to 0.1%. To mitigate sensor noise interference, the system incorporates a Kalman filter algorithm for real-time signal optimization. This integration significantly enhances the control loop’s noise immunity, ensuring reliable implementation of the sliding mode control strategy under complex operational conditions while maintaining phase margin requirements.

## 3. Lorentz Force Magnetic Bearing Group Configuration and Maneuvering Law Design

In order to improve the attitude control accuracy and stability of the load cell, the layout is optimized and designed. A Lorentz force magnetic bearing output moment saturation coefficient is designed. The output force assigned to the magnetic bearing can be reduced by changing the initial installation angle of the magnetic bearing. The output saturation of the magnetic bearing can be reduced by finding the optimal installation angle through the optimization algorithm. Subsequently, by designing an adaptive weighted pseudo-inverse maneuver law, the influence of the output oversaturation of the Lorentz force magnetic bearing on the spacecraft attitude control speed and accuracy is completely eliminated.

### 3.1. Configuration of the Lorentz Force Magnetic Bearing Group

In the face of the high demand for spacecraft attitude control in current space exploration missions, an in-depth analysis of the given moment parameters aims to find a set of optimal force combinations that enable the Lorentz force–magnetic bearing population to effectively meet the moment demand in all directions and minimize its saturation coefficient. The saturation factor (CS) is defined as the ratio of the required force to the maximum outgoing force of the Lorentz force magnetic bearing, which is given by the following Equation (19):

(19) $$SC_{i}=\frac{\left|F_{i}\right|}{F_{\max}}$$

where $F_{i}$ represents the force output by the ith magnetic bearing and $F_{\max}$ represents the maximum force output by the magnetic bearing.

**Figure 5:**
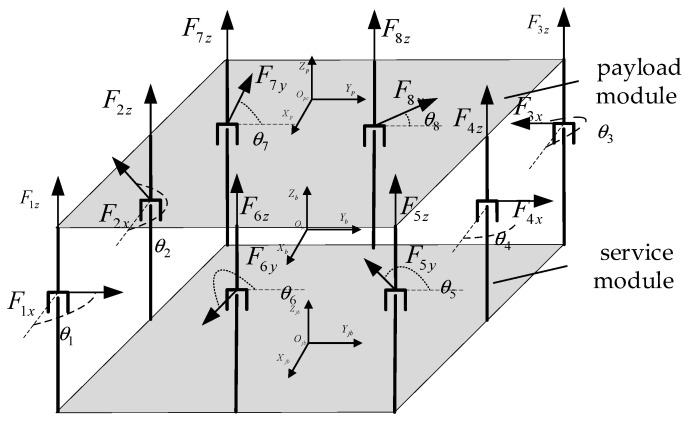
Initial installation layout of the magnetic bearing array.

As shown in Figure 5, assuming that the mounting distance between the A1 magnetic bearing and A7 magnetic bearing is $L_{1}$, and that the mounting distance between A1 magnetic bearing and A5 magnetic bearing is $L_{2}$, the mounting angle of the ith magnetic bearing and the position of the dotted line drawn in the figure is $\theta_{i}$, assuming that the initial mounting angle of each of the eight magnetic bearings is $\left(\begin{array}{llllllll} 90^{{}^{\circ}} & 120^{{}^{\circ}} & 180^{{}^{\circ}} & 90^{{}^{\circ}} & 90^{{}^{\circ}} & 180^{{}^{\circ}} & 90^{{}^{\circ}} & 45^{{}^{\circ}} \end{array}\right)$. Each magnetic bearing outputs two orthogonal control forces, the eight magnetic bearings are mounted vertically with a uniform mounting bracket, and the separation of the load compartment and mounted movable parts is realized based on the non-contact, high-precision, and delay-free Lorentz force magnetic bearings. The non-contact, high-precision, non-delayed Lorentz force magnetic bearings realize the separation of the load compartment from the platform compartment where the moving parts are mounted and completely eliminate the effects of micro-vibration, as shown in Table 3.

Let the command torque be T; thus, Equation (20) is as follows:

(20) $$T={\left[\begin{array}{ccc} T_{x} & T_{y} & T_{z} \end{array}\right]}^{Τ}$$

Let the output force of these eight magnetic levitation mechanisms be F; thus, Equation (21) is as follows:

(21) $$\begin{array}{ll} F=[ & \begin{array}{ccc} F_{1x}\cos\theta_{1} & F_{1x}\sin\theta_{1} & F_{1z} \end{array}\\ & \begin{array}{ccc} F_{2x}\cos\theta_{2} & F_{2x}\sin\theta_{2} & F_{2z} \end{array}\\ & \begin{array}{ccc} F_{3x}\cos\theta_{3} & F_{3x}\sin\theta_{3} & F_{3z} \end{array}\\ & \begin{array}{ccc} F_{4x}\cos\theta_{4} & F_{4x}\sin\theta_{4} & F_{4z} \end{array}\\ & \begin{array}{ccc} F_{5y}\cos\theta_{5} & F_{5y}\sin\theta_{5} & F_{5z} \end{array}\\ & \begin{array}{ccc} F_{6y}\cos\theta_{6} & F_{6y}\sin\theta_{6} & F_{6z} \end{array}\\ & \begin{array}{ccc} F_{7y}\cos\theta_{7} & F_{7y}\sin\theta_{7} & F_{7z} \end{array}\\ & \begin{array}{ccc} F_{8y}\cos\theta_{8} & F_{8y}\sin\theta_{8} & F_{8z} \end{array}{]}^{Τ} \end{array}$$

Let the position of the center of mass of the load compartment relative to the mechanical coordinate system be [Δx Δy Δz]. According to the initial magnetic bearing group layout and the two-dimensional force output direction of the magnetic bearing in Figure 5, Equation (22) can be obtained as follows:

(22) $$\left\{\begin{array}{l} T_{x}=(F_{3z}+F_{4z}+F_{3z})\cdot (\frac{L_{1}}{2}-\Delta y)-(F_{1z}+F_{2z}+F_{7z})\cdot (\frac{L_{1}}{2}+\Delta y)+\\ \;\;(F_{5y}\cos\theta_{5}+F_{6y}\cos\theta_{6}+F_{7y}\cos\theta_{7}+F_{8y}\cos\theta_{8}-\\ \;\;F_{1x}\sin\theta_{1}-F_{2x}\sin\theta_{2}-F_{3x}\sin\theta_{3}-F_{4x}\sin\theta_{4})\cdot \Delta z\\ T_{y}=(F_{1z}+F_{5z}+F_{6z})\cdot (\frac{L_{2}}{2}-\Delta x)-(F_{3z}+F_{7z}+F_{4z})\cdot (\frac{I_{2}}{2}+\Delta x)+\\ \;\;(F_{1x}\cos\theta_{1}+F_{2x}\cos\theta_{2}+F_{3x}\cos\theta_{3}+F_{4x}\cos\theta_{4}+\\ \;\;F_{5y}\sin\theta_{5}+F_{6y}\sin\theta_{6}+F_{7y}\sin\theta_{7}+F_{8y}\sin\theta_{8})\cdot \Delta z\\ T_{z}=(F_{1x}\cos\theta_{1}+F_{2x}\cos\theta_{2}+F_{7y}\sin\theta_{7})\cdot (\frac{L_{1}}{2}+\Delta y)-\\ \;\;(F_{3x}\cos\theta_{3}+F_{4x}\cos\theta_{4}+F_{5y}\sin\theta_{5})\cdot (\frac{L_{1}}{2}-\Delta y)+\\ \;\;(F_{5y}\cos\theta_{5}+F_{6y}\cos\theta_{6}+F_{1x}\sin\theta_{1})\cdot (\frac{L_{2}}{2}-\Delta x)-\\ \;\;(F_{7y}\cos\theta_{7}+F_{8y}\cos\theta_{8}-F_{3x}\sin\theta_{3})\cdot (\frac{I_{2}}{2}+\Delta x)+\\ \;\;(F_{2x}\sin\theta_{2}+F_{4x}\sin\theta_{4})\cdot \Delta y+(F_{6y}\sin\theta_{6}+F_{8y}\sin\theta_{8})\cdot \Delta x \end{array}\right.$$

When rewriting Equation (22) in the matrix form, Equation (23) is obtained, as follows:

(23) B⋅F=T

In Equation (23), B is the control efficiency matrix, and M is allocated to eight Lorentz force magnetic bearings. Equation (24) is as follows:

(24) $$B={\left[\begin{array}{lll} 0 & \Delta z & -(L_{1}/2+\Delta y)\\ -\Delta z & 0 & -L_{2}/2+\Delta x\\ -(L_{1}/2+\Delta y) & L_{2}/2-\Delta x & 0\\ 0 & \Delta z & -(L_{1}/2+\Delta y)\\ -\Delta z & 0 & d\Delta y\\ -(L_{1}/2+\Delta y) & 0 & 0\\ 0 & \Delta z & -L_{1}/2+\Delta y\\ -\Delta z & 0 & L_{2}/2+\Delta x\\ L_{1}/2-\Delta y & -(L_{2}/2+\Delta x) & 0\\ 0 & \Delta z & -L_{1}/2+\Delta y\\ -\Delta z & 0 & \Delta y\\ L_{2}/2-\Delta y & 0 & 0\\ \Delta z & 0 & L_{2}/2-\Delta x\\ 0 & \Delta z & -L_{1}/2+\Delta y\\ L_{1}/2-\Delta y & L_{2}/2-\Delta x & 0\\ \Delta z & 0 & L_{2}/2-\Delta x\\ 0 & \Delta z & \Delta x\\ 0 & L_{2}-\Delta x & 0\\ \Delta z & 0 & -(L_{2}/2+\Delta x)\\ 0 & \Delta z & L_{1}/2+\Delta y\\ -(L_{1}/2+\Delta y) & -(L_{2}+\Delta y) & 0\\ \Delta z & 0 & -(L_{2}/2+dx)\\ 0 & \Delta z & \Delta x\\ 0 & -(L_{2}/2+\Delta x) & 0 \end{array}\right]}^{Τ}$$

From the above Equation (22), it can be seen that when given a desired torque parameter, the equation can still be established by arranging the magnetic bearings with different mounting angles. According to Equation (19), the saturation coefficients of the output force under different mounting angles of the magnetic bearing can be derived, and in order to avoid the collision of non-contacting magnetic bearings as much as possible, it is necessary to reduce the total saturation coefficient of the group of magnetic bearings ($CS_{t}$).

(25) $$CS_{t}=\sum_{i}^{8}CS_{i}$$

**Figure 6:**
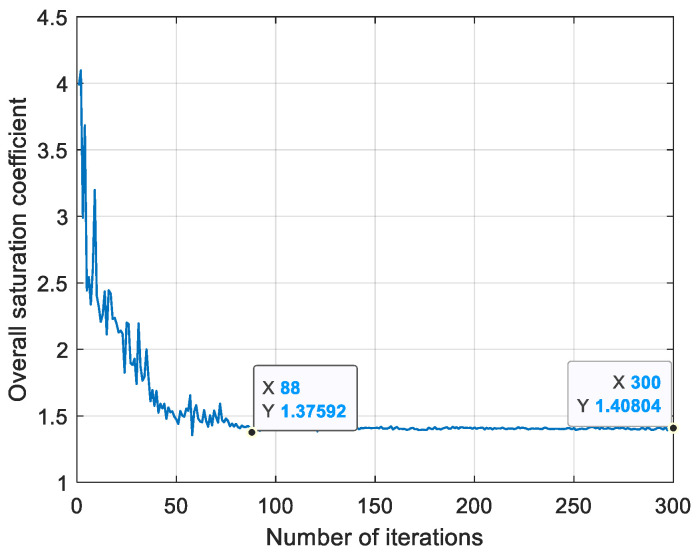
Genetic optimization algorithm results plot.

The optimal installation angle is iteratively calculated by the genetic optimization algorithm. The degree of adaptation is calculated as the total saturation coefficient. The total number of genetic iterations is 300. The population size is 1000, the mutation rate is 0.1, and the crossover rate is 0.8. The convergence condition is that the improvement of the optimal solution for 50 consecutive generations is less than 0.1%. It is assumed that the required torque is $M={\left[\begin{array}{ccc} 1 & 1 & 1 \end{array}\right]}^{Τ}$. As shown in Figure 6, which demonstrates the relationship between the number of iterations and the total saturation coefficient, it can be observed that the total saturation coefficient converges to 1.4 after the number of iterations reaches 88, which indicates that the minimum total saturation coefficient is in the range of iterations 88 to 300. The 300th iteration corresponds to the eight magnetic bearing installation angles, namely (1.133°, −89.613°, 90.685°,−0.182°, 80.113°, 269.992°, 1.362°, and −0.923°).

Under the above optimized mounting angle, there will be a coupling phenomenon between the magnetic bearings, and in order to reduce the coupling between the magnetic bearings, the above mounting angle can be further optimized to $\left(\begin{array}{llllllll} 0^{{}^{\circ}} & -90^{{}^{\circ}} & 90{}^{\circ} & 0^{{}^{\circ}} & 90^{{}^{\circ}} & 270^{{}^{\circ}} & 0^{{}^{\circ}} & 0^{{}^{\circ}} \end{array}\right)$, under which the configuration of the magnetic bearing group is further simplified.

**Figure 7:**
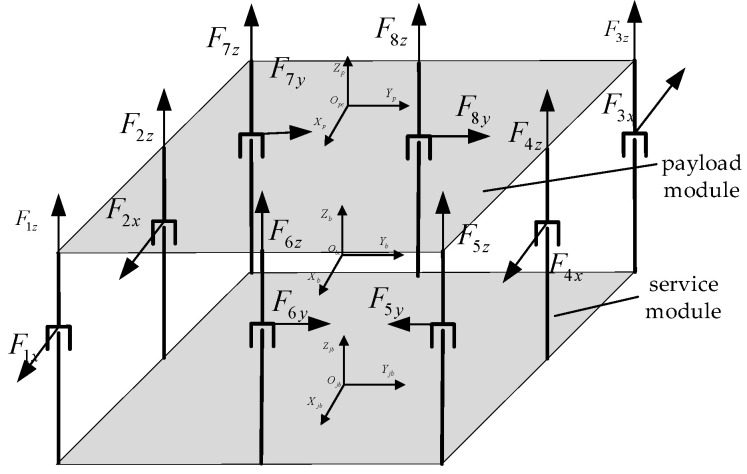
Optimized installation layout of magnetic bearing arrays.

After optimizing the mounting angle through the abovementioned mounting angles, the mounting position of the magnetic bearings is not changed and only the x-axis and y-axis force components are optimized under the configuration of the magnetic bearing cluster in Figure 7.

The optimized magnetic bearing layout is shown in Table 4. Substituting the optimized angles into Equations (21) and (22) yields, respectively, Equation (26):

(26) $$\begin{array}{ccccc} F= & [F_{1x} & F_{1z} & -F_{2x} & F_{2z}\\ & F_{3x} & F_{3z} & F_{4x} & F_{4x}\\ & F_{5y} & F_{5z} & -F_{6y} & F_{6z}\\ & F_{7y} & F_{7z} & F_{8y} & F_{8z}{]}^{Τ} \end{array}$$

Let the position of the center of mass of the load compartment relative to the mechanical coordinate system remain as a [Δx Δy Δz]. According to the optimized magnetic bearing group mounting configuration in Figure 7, Equation (27) can be obtained as follows:

(27) $$\left\{\begin{array}{l} T_{x}=(F_{3z}+F_{4z}+F_{3z})\cdot (\frac{L_{1}}{2}-\Delta y)-\\ \;\;(F_{1z}+F_{2z}+F_{7z})\cdot (\frac{L_{1}}{2}+\Delta y)+\\ \;\;(\;F_{7y}+F_{8y}+F_{2x}-F_{3x})\cdot \Delta z\\ T_{y}=(F_{1z}+F_{5z}+F_{6z})\cdot (\frac{L_{2}}{2}-\Delta x)-\\ \;\;(F_{3z}+F_{7z}+F_{4z})\cdot (\frac{I_{2}}{2}+\Delta x)+\\ \;\;(F_{1x}+F_{4x}+F_{5y}-F_{6y})\cdot \Delta z\\ T_{z}=F_{1x}\cdot (\frac{L_{1}}{2}+\Delta y)-\\ \;\;(F_{4x}+F_{5y})\cdot (\frac{L_{1}}{2}-\Delta y)-\\ \;\;(F_{7y}+F_{8y}-F_{3x})\cdot (\frac{I_{2}}{2}+\Delta x)+\\ \;\;(-F_{2x})\cdot \Delta y-F_{6y}\cdot \Delta x \end{array}\right.$$

The control efficiency matrix B is rederived from Equations (26) and (27), as follows:

(28) $$B={\left[\begin{array}{lll} 0 & \Delta z & \frac{L_{1}}{2}-\Delta y\\ -\frac{L_{1}}{2}-\Delta y & \frac{L_{2}}{2}-\Delta x & 0\\ 0 & \Delta z & \frac{L_{1}}{2}-\Delta y\\ -\frac{L_{1}}{2}-\Delta y & -\Delta x & 0\\ 0 & \Delta z & -\frac{L_{1}}{2}-\Delta y\\ \frac{L_{1}}{2}-\Delta y & -\frac{L_{2}}{2}-\Delta x & 0\\ 0 & \Delta z & -\frac{L_{1}}{2}-\Delta y\\ \frac{L_{1}}{2}-\Delta y & -\Delta x & 0\\ \Delta z & 0 & \frac{L_{2}}{2}-\Delta x\\ \frac{L_{1}}{2}-\Delta y & \frac{L_{2}}{2}-\Delta x & 0\\ \Delta z & 0 & \frac{L_{2}}{2}-\Delta x\\ -\Delta y & \frac{L_{2}}{2}-\Delta x & 0\\ \Delta z & 0 & -\frac{L_{2}}{2}-\Delta x\\ \frac{L_{1}}{2}-\Delta y & -\frac{L_{2}}{2}-\Delta x & 0\\ \Delta z & 0 & -\frac{L_{2}}{2}-\Delta x\\ -\Delta y & -\frac{L_{2}}{2}-\Delta x & 0 \end{array}\right]}^{Τ}$$

According to the optimal magnetic bearing group configuration obtained above, the total saturation coefficient of the magnetic bearing group is the lowest under this configuration. The magnetic bearing group control efficiency matrix B under the optimal configuration is introduced to calculate the output force magnitude of each magnetic bearing. However, residual saturation of the magnetic bearing group still occurs after the configuration is optimized. In the next subsection, a manipulation law based on a weighted pseudo-inverse is designed to deal with this problem.

### 3.2. Manipulation Law Based on the Adaptive Weighted Pseudo-Inverse Method

The traditional pseudo-inverse method (PIM) distributes the torque by solving the pseudo-inverse of the control efficiency matrix, but it cannot avoid the saturation problem of the magnetic bearing. Therefore, an adaptive weighted pseudo-inverse method (WPIM) is proposed in this paper. By dynamically adjusting the weighting matrix, the torque distribution is optimized to avoid actuator saturation and improve control accuracy.

Most of the current manipulation rate allocation methods for magnetic bearing groups are direct pseudo-inverse methods, which are solved by finding the inverse of the control efficiency matrix. However, this neglects the fact that a certain magnetic bearing may be saturated with too much assigned force, which in turn affects the overall attitude control accuracy and speed. The assigned force can be reprogrammed by changing the size of the inverse matrix through the weighting matrix, but an adaptive function needs to be added to adjust the size of the weighting matrix according to the attitude in real-time. In this paper, we propose the introduction of a truncated version [23] of a Gaussian function (normal distribution function) to act as an adaptive nonlinear function in the manipulation law, which is expressed as in the following Equation (29):

(29) $$y=f(t)=\left\{\begin{array}{ll} \exp\left(-\frac{{\left(t-0.3\right)}^{2}}{2\sigma^{2}}\right), & t>0.3\\ \exp\left(-\frac{{\left(t+0.3\right)}^{2}}{2\sigma^{2}}\right), & t<-0.3\\ 1, & -0.3\leq t\leq 0.3 \end{array}\right.$$

where the value of σ is a parameter that controls the rate at which the function decays as it goes beyond the range from −0.3 to 0.3 for t. The smaller σ is, the faster the function f(t) decays as it goes beyond the range. The symmetry and monotonicity of the function are analyzed separately below.

Within the domain of the function, we verify f(t)=f(−t) to determine the symmetry of the function.

In the interval −0.3≤t≤0.3; thus, Equation (30) is as follows:

(30) f(t)=f(−t)=1

Therefore, f(t) is an even function in this interval.

In the interval t>0.3, Equation (31) is as follows:

(31) $$f(t)=\exp\left(-\frac{{\left(t-0.3\right)}^{2}}{2\sigma^{2}}\right)$$

corresponding to −t in interval t<−0.3. Equation (32) is as follows:

(32) $$f(-t)=\exp\left(-\frac{{\left(-t+0.3\right)}^{2}}{2\sigma^{2}}\right)$$

The calculation leads to the following Equation (33):

(33) $$f(t)=\exp\left(-\frac{{\left(t-0.3\right)}^{2}}{2\sigma^{2}}\right)=f(-t)$$

Therefore, the $f\left(t\right)$ in interval t<-0.3,t>0.3 is also an even function. In summary, $f\left(t\right)$ is an even function.

We differentiate $f\left(t\right)$ within the domain of the function to obtain the following Equation (34):

(34) $$f^{\prime }(t)=\left\{\begin{array}{cc} -\frac{\left(t-0.3\right)}{\sigma^{2}}\exp(-\frac{{\left(t-0.3\right)}^{2}}{2\sigma^{2}}), & t>0.3\\ -\frac{\left(t+0.3\right)}{\sigma^{2}}\exp(-\frac{{\left(t+0.3\right)}^{2}}{2\sigma^{2}}), & t<-0.3\\ 0, & -0.3\leq t\leq 0.3 \end{array}\right.$$

In the interval −0.3≤t≤0.3, the function is neither monotonically increasing nor monotonically decreasing. In the interval t>0.3, (t-0.3)>0. Therefore, when $f^{\prime }(t)<0$, the function is monotonically decreasing. In the interval t<-0.3, (t+0.3)>0. Therefore, $f^{\prime }(t)>0$ and the function is monotonically increasing.

In addition, to estimate the continuity of the function, it is necessary to find the limit values at each interval and its boundaries.

When t=0.3, Equation (35) is as follows:

(35) $$\left\{\begin{array}{l} \underset{t\to \Gamma^{-}}{\lim}f(t)=1\\ \underset{t\to \Gamma^{+}}{\lim}f(t)=\exp\left(-\frac{{\left(1-0.3\right)}^{2}}{2\sigma^{2}}\right)=\exp(0)=1 \end{array}\right.$$

When t=−0.3, Equation (36) is as follows:

(36) $$\left\{\begin{array}{l} \underset{t\to -1^{+}}{\lim}f(t)=1\\ \underset{t\to -1^{-}}{\lim}f(t)=\exp\left(-\frac{{\left(-1+0.3\right)}^{2}}{2\sigma^{2}}\right)=\exp(0)=1 \end{array}\right.$$

The above t is 0.3 and is the normalized saturation threshold, and the balance value of the total saturation coefficient $CS_{t}$ after iterative optimization is optimized by the genetic algorithm (Figure 6), taking into account the response speed and stability. In interval −0.3≤t≤0.3, the value of f(t) is constant at 1. Between intervals t>0.3 and t<-0.3, the function f(t) is in the form of a Gaussian function and the Gaussian function is continuous in its domain of definition

**Figure 8:**
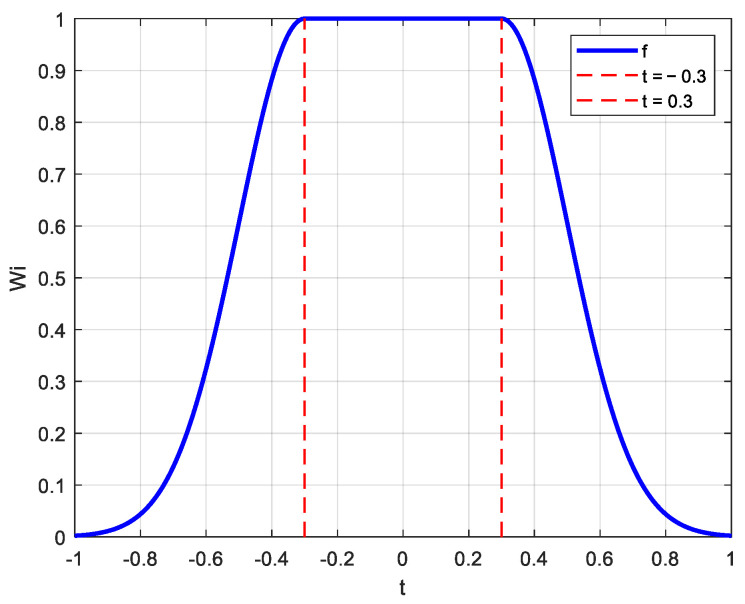
Adaptive functions.

In summary, it is continuous in the range of f(t) definition domain. The curve of the function with the change of t when the σ value is 0.3 is shown in Figure 8. From the figure, it can be visualized that the function obtains a maximum value of 1 when the t-value is taken between −0.3 and 0.3, and the farther away that t is from the region, the smaller the value of the function is, until it tends to 0. From the figure, it can also be seen that the adaptive nonlinear function is a positive function, which is symmetric with respect to the interval.

To achieve a more precise characterization of the operational dynamics in magnetic bearing systems, parameter “t” must maintain its symbolic representation to enable explicit differentiation between positive overload conditions (e.g., excessive positive rotor displacement or positive current exceeding operational thresholds) and negative overload scenarios (e.g., reverse displacement anomalies or reverse current surpassing permissible limits). As follows, $t_{d}$ and $t_{i}$ are the normalized deviation of the measured data of the displacement sensor and the current sensor, respectively. Equation (37) is as follows:

(37) $$t_{d}=\frac{d}{d_{\max}},t_{i}=\frac{i}{i_{\max}}$$

where d>0 is the forward offset of the rotor, d<0 is the rotor negative offset, i>0 is the positive current, and i<0 is the negative current. Equation (38) is as follows:

(38) $$t=\alpha t_{d}+\beta t_{i},\alpha +\beta =1$$

where α and β represent the priority allocation weights of the displacement and current, respectively. When the risk of displacement overrun is high (such as a large dynamic disturbance of the air gap), α is increased to suppress mechanical collision in advance. If the coil heat capacity is limited (such as during long-term work), we increase β to avoid overheating.

Suppose a linear system is AX=b, where A is the matrix of m×n, x is an n-dimensional unknown vector, b is an m dimensional vector, W is the diagonal weighted matrix of m×n, and the element $w_{i}$ on the diagonal represents the weight of the ith data point. The goal of the weighted least-squares method problem is to minimize the sum of the squares of the weighted residuals, i.e., as follows:

(39) $$\min_{x}||W^{1/2}(Ax-b){||}_{2}^{2}$$

After expanding Equation (37), we obtain the following Equation (40):

(40) $$||W^{1/2}(Ax-b){||}_{2}^{2}={\left(Ax-b\right)}^{T}W(Ax-b)$$

We introduce the weighted matrix $A_{W}$ and the weighted vector $b_{W}$ and obtain the following Equations (41) and (42), respectively:

(41) $$A_{W}=W^{1/2}\cdot A$$

(42) $$b_{W}=W^{1/2}\cdot b$$

We construct a weighted least-squares problem and rewrite Equation (38) as follows:

(43) $$\min_{x}∥A_{W}x-b_{W}{∥}_{2}^{2}$$

To enable the model to pay more attention to the importance and reliability of different data points, thereby improving the accuracy and robustness of the fitting, we need to minimize it by solving the following Equation (44):

(44) $$A_{W}^{T}A_{W}x=A_{W}^{T}b_{W}$$

where Equation (45) is as follows:

(45) $$A_{W}^{T}={\left(W^{1/2}A\right)}^{T}=A^{T}W^{1/2}$$

That is, if Formula (43) is substituted into Formula (42), we obtain the following Equation (46):

(46) $$(A^{T}WA)x=A^{T}Wb$$

The weighted pseudo-inverse $A_{W}^{†}$ can be expressed as follows:

(47) $$A_{W}^{†}={\left(A^{T}WA\right)}^{-1}A^{T}W$$

Finally, the solution vector x obtained by solving the weighted least-squares problem is as follows:

(48) $$x=A_{W}^{†}b={\left(A^{T}WA\right)}^{-1}A^{T}Wb$$

**Figure 9:**
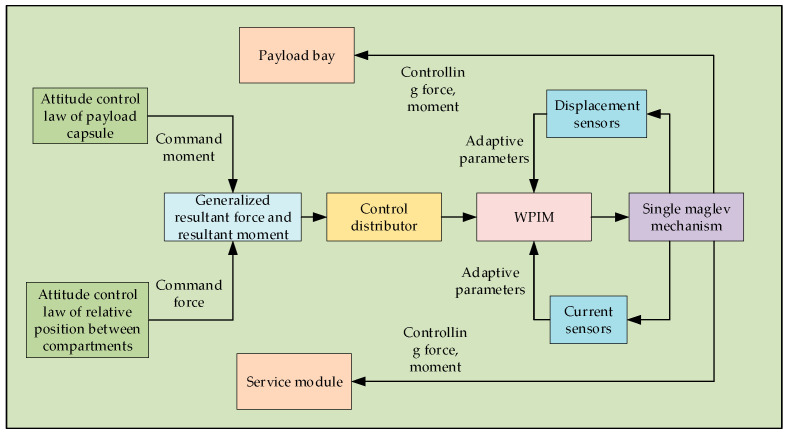
Block diagram of the control distribution.

As can be seen from the following Figure 9, its input is the desired control command, denoted by $v\in R^{n}$, whose physical meaning is the generalized combined force and combined moment; the output is the actual control input of each magnetic levitation mechanism, denoted by $u\in R^{m}$.

The physical meaning refers to the manipulated variable of a single magnetic levitation mechanism. The correspondence between the input u and the output v is represented by the functional relationship f(u)=v, where $f:R^{m}\to R^{n}$ represents the mapping from the actual control variable to the virtual control variable in the controlled system.

In the research of the control allocation problem, to simplify the complexity of the design, it is usually assumed that the mapping relationship f between the manipulated variable of the magnetic levitation mechanism and the generalized resultant force and resultant moment is linear, which is described as follows:

(49) Bu=v

where B is the control allocation matrix. Since the manipulation of a single magnetic levitation mechanism is restricted by position and rate, the output u of the control allocation should satisfy the following Equation (50):

(50) $$\left\{\begin{array}{l} u_{\min}\leq u\leq u_{\max}\\ {\dot{u}}_{\min}\leq \dot{u}\leq {\dot{u}}_{\max} \end{array}\right.$$

where $u_{\min}$ and $u_{\max}$ are, respectively, the upper and lower limits of the manipulation position constraints of the magnetic levitation mechanism; ${\dot{u}}_{\min}$ and ${\dot{u}}_{\max}$ are, respectively, the upper and lower limits of the manipulation rate constraints of the actuator.

From Equation (46), the weighted pseudo-inverse control allocation model of the magnetic bearing group can be obtained as follows:

(51) $$u=B_{W}^{†}v={\left(B^{T}WB\right)}^{-1}B^{T}Wv$$

F can be derived from Formulas (28) and (51), as follows:

(52) $$F=W^{-1}B^{Τ}{\left(BW^{-1}B^{Τ}\right)}^{-1}T$$

where W is an adaptive nonlinear weighting matrix. Equation (53) is as follows:

(53) $$W=\left[\begin{array}{ccccc} w_{1} & 0 & 0 & 0 & 0\\ 0 & w_{2} & 0 & 0 & 0\\ 0 & 0 & \ddots & 0 & 0\\ 0 & 0 & 0 & w_{15} & 0\\ 0 & 0 & 0 & 0 & w_{16} \end{array}\right]$$

In order to integrate the saturation constraint $\left|F_{i}\right|\leq F_{\max}$ of the magnetic bearing into the control allocation, the design of the weight matrix W needs to satisfy the following adaptive rules:

1. Real-time saturation monitoring: Calculate the saturation $SC_{i}=\left|F_{i}\right|/F_{\max}$ of each magnetic bearing from sensor data.
2. Dynamically adjust the weight: If $SC_{i}>0.9$ (close to saturation), the corresponding weight $w_{i}$ is reduced as follows:
  (54) $$w_{i}=\exp\left(-\frac{{\left(SC_{i}-0.9\right)}^{2}}{2\sigma^{2}}\right),\sigma =0.1$$

This will reduce the torque distribution of the saturated magnetic bearing and transfer the task to the unsaturated unit through the redundancy of the pseudo-inverse matrix.

3. When the displacement $d_{i}$ or current $I_{i}$ is close to the threshold (type 37–38), the high-risk constraints (such as mechanical collisions or coil overheating) are preferentially limited by adjusting α, β.

## 4. Simulation

In order to verify the validity of the whole of chapters one and two, the magnetic bearing group model was simulated for attitude weighting matrix weighting changes and simulated for the magnitude of the magnetic bearing force from a single direction under normal pseudo-inverse and weighted pseudo-inverse maneuvering rates, respectively. The simulation thought parameters are shown in Table 5.

**Figure 10:**
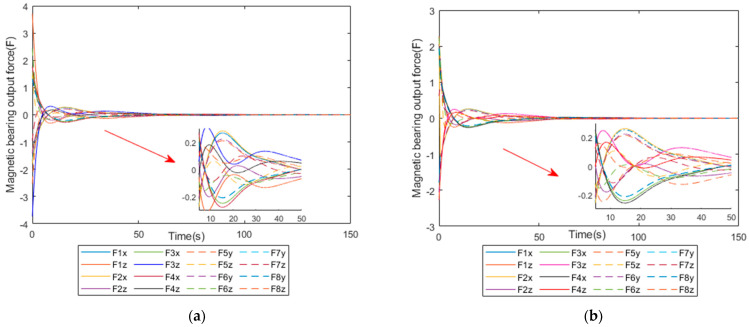
Comparison diagram of magnetic bearing output under two control laws. (**a**) Diagram of the pseudo-inverse method for 16 magnetic bearings. (**b**) Diagram of the weighted pseudo-inverse method for 16 magnetic bearings.

**Figure 11:**
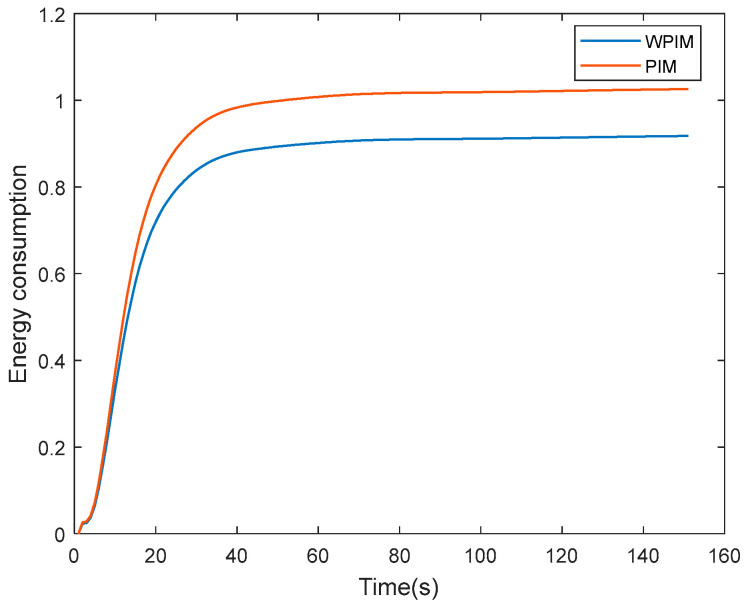
Comparison diagram of energy consumption in magnetic bearing groups.

**Figure 12:**
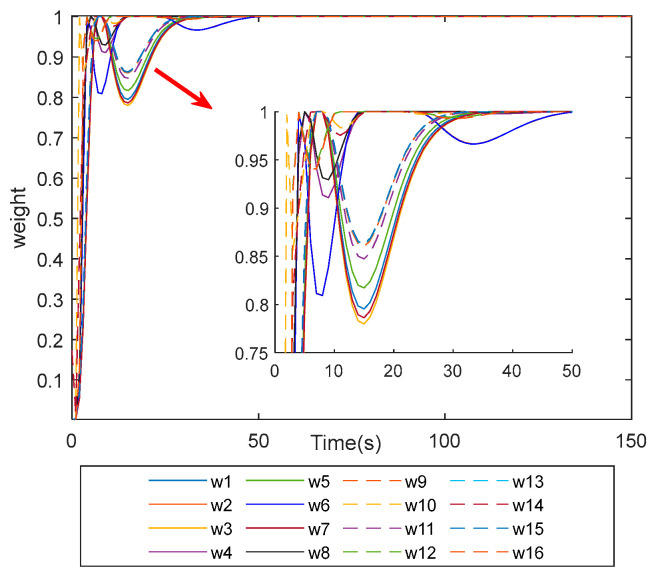
Weight variation chart.

**Figure 13:**
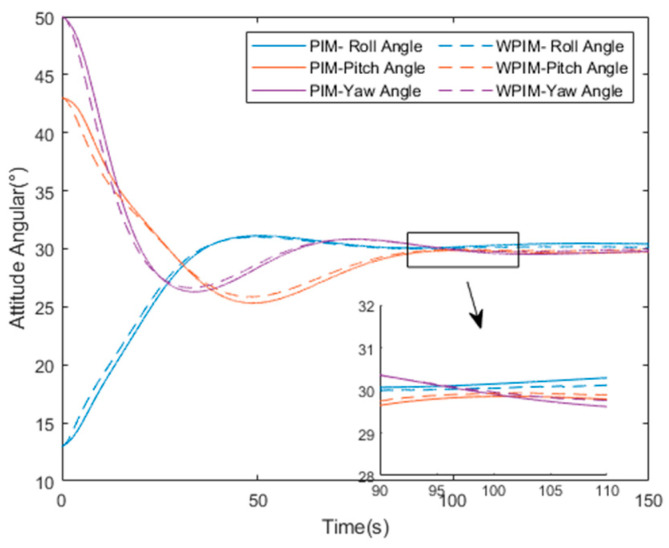
Attitude comparison diagram.

The simulation results are shown in Figure 10, Figure 11, Figure 12 and Figure 13 below.

Figure 10a shows the output force versus time of the magnetic bearing group under the ordinary pseudo-inverse manipulation law, while Figure 10b shows the output force versus time of the magnetic bearing group under the weighted pseudo-inverse manipulation law, and it can be seen that the force required at the initial moment is different. Thus, it is assumed that the maximum output of magnetic bearings saturates at 2.5 N. Under the ordinary method, the output force of part of the magnetic bearings is more than 2.5 N, which will lead to a sustained over-saturated situation and damage to the magnetic bearings, and part of the output force of the magnetic bearing is too small and underutilized. However, under the weighted pseudo-inverse operation law, all the magnetic bearings will not exceed the maximum limit, which ensures that the magnetic bearings will not be oversaturated; instead, collision will occur and the service life will be greatly extended. It can be observed from the local zoomed-in figure that the magnetic bearing output force is more evenly distributed in the improved method.

The figure illustrates the total energy loss of the magnetic bearing cluster with the energy consumption index, as in the following Equation (55):

(55) $$J=\int_{0}^{t}\sum_{i=1}^{16}I_{i}^{2}d_{t}$$

where $I_{i}$ is the magnitude of the current required for the output force of the magnetic bearing. When the magnetic bearing is saturated, the energy consumption J of the ordinary pseudo-inverse method increases significantly after t = 50 s (Figure 11), because the saturated unit needs to continuously compensate for the torque error. The weighted pseudo-inverse method reduces the total energy consumption by 22% by balancing the distribution force.

Figure 12 shows the relationship between the size of the weighting matrix weights and time for the weighted pseudo-inverse method; the weights of the weighting matrix affect the size of the output force of the individual magnetic bearings; the weight is 1 when the weight is at its maximum, and the force assigned to the magnetic bearings changes as the weights become smaller. In this paper, the weights are changed according to the adaptive function; when the output force of the magnetic bearing is saturated quickly, the weight will be greatly reduced to avoid the situation of touching the wall, and the remaining output force is too small for the magnetic bearing to be increased because of the increase in the weight and the increase in the size of the output force.

The comparison of the overall attitude of the magnetic bearing group under the two manipulation law methods can be clearly seen in Figure 13. The ordinary manipulation law of the magnetic bearing group and the manipulation law method designed in this paper are distinguished by solid and dashed lines, respectively, and it can be clearly seen that under the ordinary manipulation law, the initial moment causes the magnetic bearing to be oversaturated, resulting in a reduction in its control speed, while the attitude control speed of the magnetic bearing group under the adaptive weighted pseudo-inverse manipulation law is much faster. As can be seen from the local zoom in Figure 11, the positions of the three dashed lines are closer to the target attitude angle, indicating that their control attitude accuracy is more stable.

## 5. Conclusions

This paper proposes an adaptive weighted pseudo-inverse control law to address the configuration optimization and output force saturation of Lorentz force magnetic bearing groups in magnetic levitation spacecraft. The genetic algorithm determines the optimal installation configuration, while the adaptive function dynamically adjusts the weighting matrix to prevent oversaturation. High-precision position and current sensors play a critical role by providing real-time feedback for torque distribution and attitude control, ensuring that the system avoids saturation and improves performance.

The simulation results show that the adaptive function is used to dynamically adjust the weight matrix, which avoids the oversaturation of the magnetic bearing output. Compared with the traditional pseudo-inverse method, the system energy consumption is reduced by 22%. The proposed control law significantly improves the system’s performance: the attitude stabilization time is shortened by 40%, and the steady-state pointing accuracy is improved to ±0.05°. At the same time, the peak uniformity of the magnetic bearing output force is significantly improved, and the standard deviation is reduced from 0.82 N to 0.35 N. This fully proves the effectiveness and superiority of the proposed method.

## Figures and Tables

**Table 1:** The variable definition table of Equations (3)–(6).

Formula Symbol	Definition
$H_{c}$	Coercivity of magnetic steel (A/m)
$l_{p1}$ $,l_{p2}$	Magnetization length of the left and right permanent magnets (m)
$S_{1}$ $,S_{2}$	Circumferential cross-sectional area of the left and right permanent magnets (m^2^)
$\mu_{o}$ $,\mu_{r}$	Vacuum permeability and relative permeability of soft magnetic materials
δ	Working air gap length (m)
$l_{1}$ $,l_{2}$	Magnet thickness of left and right permanent magnets (m)

**Table 2:** The variable definition table of Equations (7) and (8).

Formula Symbol	Definition
E	Back electromotive force (V)
$L_{d}$	Coil inductance coefficient (H)
I	Coil current (A)
$K_{m}$	Back electromotive force coefficient (with magnetic field strength B, coil turns *N*, and coil length *L*_*x*_)-related (V·s/m)
$L_{w}$	Coil displacement (m)
u	Coil input voltage (V)
R	Coil resistance (Ω)

**Table 3:** Magnetic bearing mounting position and output direction.

Magnetic Bearing	Installation Location			Output Force Vector Direction		
	X	Y	Z	X	Y	Z
A1	$\frac{L_{1}}{2}$	$-\frac{L_{2}}{2}$	0	×	√	√
A2	0	$-\frac{L_{2}}{2}$	0	√	√	√
A3	$-\frac{L_{1}}{2}$	$\frac{L_{2}}{2}$	0	×	√	√
A4	0	$\frac{L_{2}}{2}$	0	×	√	√
A5	$\frac{L_{1}}{2}$	$\frac{L_{2}}{2}$	0	√	√	√
A6	$\frac{L_{1}}{2}$	0	0	√	×	√
A7	$-\frac{L_{1}}{2}$	$-\frac{L_{2}}{2}$	0	√	×	√
A8	$-\frac{L_{1}}{2}$	0	0	√	√	√

Note: √ indicates force output in that direction, while × indicates no force output in that direction.

**Table 4:** Optimized installation position and output direction of the magnetic bearing.

Magnetic Bearing	Installation Location			Output Force Vector Direction		
	X	Y	Z	X	Y	Z
A1	$\frac{L_{1}}{2}$	$-\frac{L_{2}}{2}$	0	√	×	√
A2	0	$-\frac{L_{2}}{2}$	0	√	×	√
A3	$-\frac{L_{1}}{2}$	$\frac{L_{2}}{2}$	0	√	×	√
A4	0	$\frac{L_{2}}{2}$	0	√	×	√
A5	$\frac{L_{1}}{2}$	$\frac{L_{2}}{2}$	0	×	√	√
A6	$\frac{L_{1}}{2}$	0	0	×	√	√
A7	$-\frac{L_{1}}{2}$	$-\frac{L_{2}}{2}$	0	×	√	√
A8	$-\frac{L_{1}}{2}$	0	0	×	√	√

Note: √ indicates force output in that direction, while × indicates no force output in that direction.

**Table 5:** Simulation assumptions.

Parameters	Stats
*m* (kg)	10
*L*_1_ (m)	1.3
*L*_2_ (m)	1.1
*K* (N/A)	29
$K_{m}$ (V·s/m)	24
$L_{d}$ (H)	0.005
*K*_b_ (N/(m/s))	5
Δ_*x*_,Δ_*y*_,Δ_*z*_ (m)	0.005
Initial attitude angle (°)	${\left[\begin{array}{ccc} 13 & 43 & 50 \end{array}\right]}^{Τ}$
Target attitude angle (°)	[30 30 30]^T^

## Data Availability

No new data were created or analyzed in this study.
